# The Inflammatory Gene *PYCARD* of the Entorhinal Cortex as an Early Diagnostic Target for Alzheimer’s Disease

**DOI:** 10.3390/biomedicines11010194

**Published:** 2023-01-12

**Authors:** Wenjia Liu, Sophia Chen, Xin Rao, Yisong Yang, Xiaodong Chen, Liyang Yu

**Affiliations:** 1 School of Electronics and Information, Hangzhou Dianzi University, Hangzhou 310018, China; 2 School of Medicine, Imperial College London, London SW7 2AZ, UK; 3 School of Electronic Engineering and Computer Science, Queen Mary University of London, London E1 4NS, UK

**Keywords:** Alzheimer’s disease, entorhinal cortex, inflammation, differentially expressed gene, *PYCARD*, diagnostic target

## Abstract

The incidence of Alzheimer’s disease (AD) is increasing year by year, which brings great challenges to human health. However, the pathogenesis of AD is still unclear, and it lacks early diagnostic targets. The entorhinal cortex (EC) is a key brain region for the occurrence of AD neurodegeneration, and neuroinflammation plays a significant role in EC degeneration in AD. This study aimed to reveal the close relationship between inflammation-related genes in the EC and AD by detecting key differentially expressed genes (DEGs) via gene function enrichment pathway analysis. GSE4757 and GSE21779 gene expression profiles of AD were downloaded from the Gene Expression Omnibus (GEO) database. R language was used for the standardization and differential analysis of DEGs. Then, significantly enriched Gene Ontology (GO) terms and Kyoto Encyclopedia of Genes and Genomes (KEGG) pathways were analyzed to predict the potential biological functions of the DEGs. Finally, the significant expressions of identified DEGs were verified, and the therapeutic values were detected by a receiver operating characteristic (ROC) curve. The results showed that eight up-regulated genes (*SLC22A2*, *ITGB2-AS1*, *NIT1*, *FGF14-AS2*, *SEMA3E*, *PYCARD*, *PRORY*, *ADIRF*) and two down-regulated genes (*AKAIN1*, *TRMT2B*) may have a potential diagnostic value for AD, and participate in inflammatory pathways. The area under curve (AUC) results of the ten genes showed that they had potential diagnostic value for AD. The AUC of *PYCARD* was 0.95, which had the most significant diagnostic value, and it is involved in inflammatory processes such as the inflammasome complex adaptor protein. The DEGs screened, and subsequent pathway analysis revealed a close relationship between inflammation-related *PYCARD* and AD, thus providing a new basis for an early diagnostic target for AD.

## 1. Introduction

Alzheimer’s disease (AD) is a common progressive degenerative disease affecting the brain, characterized by global impairment of cognition, function, and behavior. AD has the highest incidence in the elderly population, with the prevalence approximately doubling every five years after the age of 65 [1,2,3]. With the global increase in human life expectancy and the ageing population, the prevalence of AD is also increasing, which poses huge challenges to modern society via issues such as healthcare and social care costs [4]. Neuropathological characteristics of AD patients include intracellular neurofibrillary tangles (NFTs) composed of hyperphosphorylated microtubule-associated protein tau, and extracellular amyloid plaques formed from the beta-amyloid (Aβ) peptides [5,6]. The entorhinal cortex (EC), a key brain region where AD-related neurodegeneration occurs, is the first region affected, before AD spreads to other brain regions [7]. Although thinning of the nasal cortex and atrophy of the hippocampus have also been observed in AD [8,9], anterolateral EC thinning occurs very early in AD, and is associated with upregulation of amyloid and tau proteins in cerebrospinal fluid (CSF) [10]. Consequently, changes in the EC are more likely to predict the development of AD than hippocampal atrophy [11], and these early changes in EC could make it an early indicator of AD.

Moreover, on a cellular level, the EC in AD demonstrates a unique expression pattern of glial proteins, with selective dysfunction and neuroinflammation potentially contributing to the early degeneration of EC neurons [12]. Glial cells (astrocytes and microglia) are involved in a variety of biological activities, including neuronal structural support and nutrient provision, maintenance of intercellular homeostasis and ion gradients, clearance of synaptic neurotransmitters, mediation of immune responses, and reduction of oxidative stress [13,14,15,16,17]. In addition, glial cells can also release inflammatory molecules to maintain cell integrity. During chronic inflammation, abnormally activated astrocytes and microglia can cause neuronal death by releasing reactive oxygen species (ROS) and nitrifying molecules [12,18]. Perturbation of glial cells and neuroinflammatory changes have been mentioned several times in the EC of AD brain, in which glial dysregulation and pro-inflammatory molecules play a central role in the process of AD degeneration [19,20,21,22]. For example, increased immune expression levels of the proinflammatory molecule glia maturation factor (GMF) activated astrocytes and microglia, and these phenomena are significantly clustered at sites of amyloid plaques and NFTs [22]. Yeh et al. analyzed astrocyte morphology in the EC of an AD transgenic mice model (3xTg-AD), and the results showed that the surface and volume of astrocytes were reduced in the 1-month mice compared with the control group [23]. In addition, early up-regulation of tumor necrosis factor (TNF-α) and monocyte chemoattractant protein (MCP-1) were observed in the EC of 3xTg-AD mice, which was associated with an increase in microglia and macrophages, suggesting a correlation between EC and inflammation [21]. Aβ also activates microglial cell receptors and triggers advanced glycation end products that may directly contribute to the degeneration of EC neurons, and the progression of synaptic and behavioral defects [20]. Therefore, studying the key role of neuroinflammation in the EC can help identify new determinants in AD progression [24,25,26], and provide new strategies for early detection of AD or future treatment to control AD.

Gene expression profiling is a well-documented and established method for determining causative factors associated with the occurrence and progression of disease. Several studies have identified potential gene and specific changes associated with AD pathology using microarray. For example, GSE26972 (three AD patients and three control subjects) was used to screen the differentially expressed genes (DEGs) of AD [27]. Wu et al. identified key immunological genes associated with AD by GSE110226 (seven AD patients and six control subjects) and GSE122063 (twelve AD patients and eleven control subjects) [28]. Based on two gene expression microarrays (GSE63060, 145 AD patients and 104 control subjects; GSE63061, 140 AD patients and 135 control subjects) in peripheral blood and one gene methylation microarray (GSE153712, 161 AD patients and 471 control subjects), Qiu et al. identified the key differential methylation and DEGs of AD [29]. Additionally, several regulatory factors and biomarkers in microglia were identified using GSE65067 (three wild-type and five AD mice) [30], and gene expression profiles of laser-captured EC neurons from postmortem AD identified specific changes that triggered a pathological cascade of events in AD [31]. The key stimuli leading to the development of AD remain unclear, but gene expression in postmortem brain tissue may reveal pathways involved in the development of AD [32].

Since the EC is located in the medial part of the temporal cortex, in this study, the gene expression profiles of the human EC GSE4757 and the temporal cortex GSE21779 were selected in this study. Through the detection of DEGs, as well as pathway analysis between AD patients and a control group, the close relationship between inflammation related genes in the EC and AD was revealed. The significant expressions of identified DEGs were then verified, and the diagnostic values were detected by the receiver operating characteristic (ROC) curve. This study provides a novel basis for the correlation between inflammation and AD, which is of great significance for the early diagnosis and treatment of AD.

## 2. Methods

### 2.1. Data Download and Preprocessing

The Gene Expression Omnibus (GEO) database (http://www.ncbi.nlm.nih.gov/geo/ (accessed on 5 January 2023)) is a public functional genomics data repository and mainly based on microarray data. GSE4757 [33] and GSE21779 [34] gene expression profiles (GPL570: Affymetrix human genome chips U133 Plus 2.0 Array) were downloaded from the GEO database. The GSE4757 expression profile included 10 AD patients and 10 control subjects (relevant age and gender unknown); GSE21779 expression profile included 2 AD patients (2 older females) and 16 control subjects (4 older males and 7 females, 1 young male and 4 females) (exact age unknown). Each sample in the expression profile contained more than 54,000 genetic data.

### 2.2. Identification of Differentially Expressed Genes (DEGs) for AD

Standardization and differential analysis of differentially expressed genes (DEGs) between AD and the healthy samples were analyzed using R language 3.2.3 (“limma” package) with *p* value < 0.05 and |logFC| > 1.0 (FC, fold change) [35].

### 2.3. Functional Enrichment Analysis of DEGs

Gene Ontology (GO) function and enrichment analysis of Kyoto Encyclopedia of Genes and Genomes (KEGG) pathways were performed through R language (“org.Hs.eg.db” package and “clusterProfiler” package) [36]. The GO terms and KEGG pathways with *p* value < 0.05 were identified.

### 2.4. Verification of the DEGs

The expressions of identified DEGs were further verified, and the receiver operating characteristic (ROC) analysis was performed using R language (“survivalROC” package) to test the therapeutic value of these DEGs [37].

## 3. Results

### 3.1. Screening of DEGs

**Figure 1:**
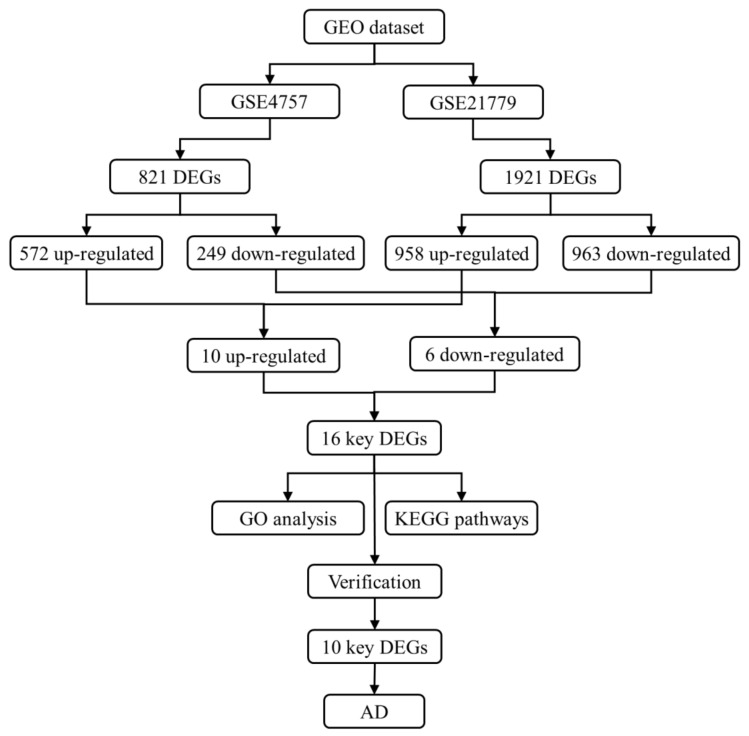
The analysis flow chart of this study. DEGs: differentially expressed genes; GO: gene ontology; KEGG: Kyoto Encyclopedia of Genes and Genomes; AD: Alzheimer’s disease.

**Figure 2:**
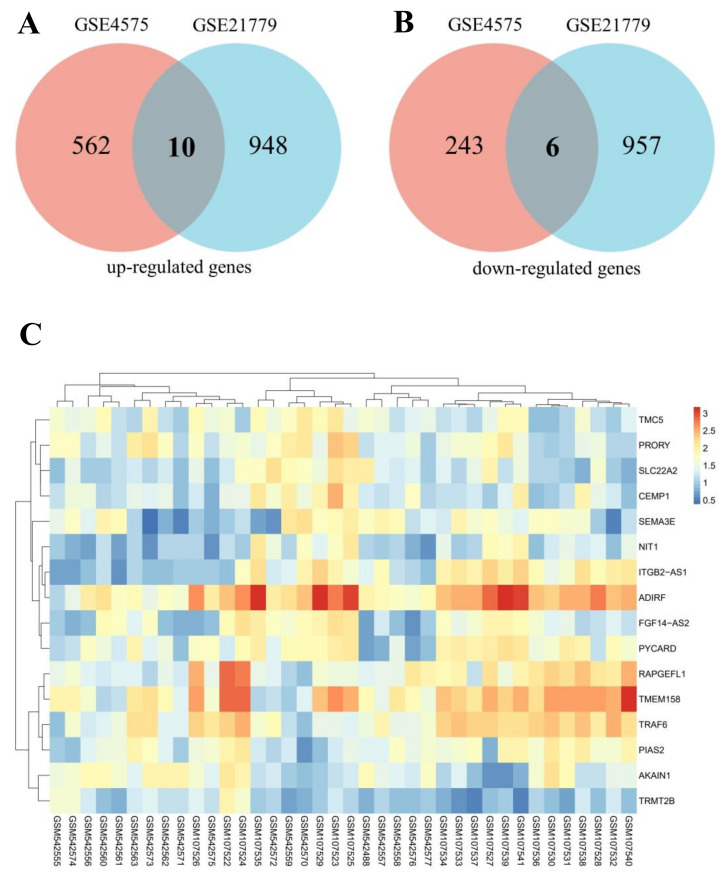
Identification of DEGs. (**A**) The Venn diagram of 10 up-regulated genes; (**B**) The Venn diagram of 6 down-regulated genes; The pink represents GSE4575 dataset and blue represents GSE21779 dataset; (**C**) The heatmap image of 16 DEGs.

The analysis flow chart of this study is demonstrated in Figure 1. AD patients and control subjects had differential gene analysis performed. The GSE4757 and GSE21779 expression profiles displayed 821 DEGs (572 up-regulated and 249 down-regulated) and 1921 DEGs (958 up-regulated and 963 down-regulated), respectively. Then, 16 key DEGs were obtained through the intersection of DEGs from GSE4757 and GSE21779, including 10 up-regulated genes and 6 down-regulated genes (Table 1). Venn diagrams of the up-regulated and down-regulated genes are shown in Figure 2A,B, and the heatmap is shown in Figure 2C.

### 3.2. DEGs Functional Enrichment Analysis

**Figure 3:**
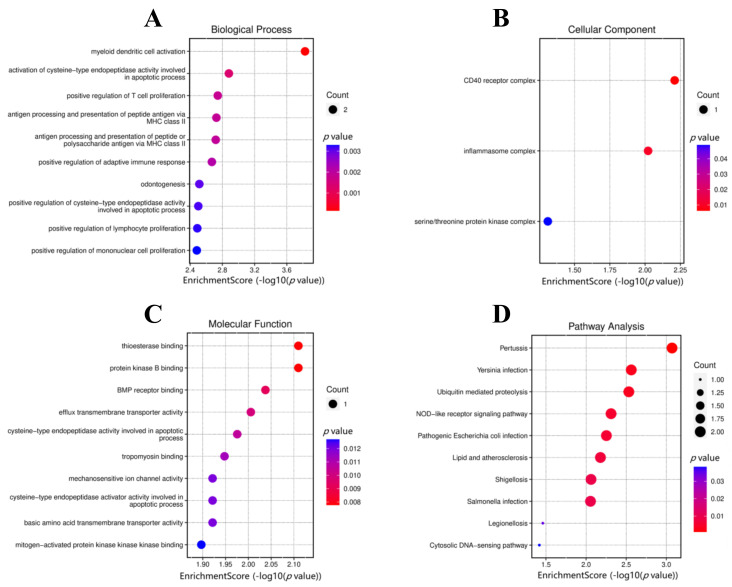
Functional enrichment analysis of target genes. (**A**) BP; (**B**) CC; (**C**) MF; (**D**) KEGG enrichment pathways of DEGs.

The GO enrichment analysis of DEGs, including biological process (BP), cellular component (CC), and molecular function (MF) are shown in Table 2, and visualized by dot plot (Figure 3A–C). BP mainly contained myeloid dendritic cell activation, activation of cysteine-type endopeptidase activity involved in the apoptotic process, and positive regulation of T cell proliferation. CC mainly contained the CD40 receptor complex, inflammasome complex, and serine/threonine protein kinase complex. MF mainly contained thioesterase binding, protein kinase B binding, and BMP receptor binding. The KEGG pathway analysis revealed multiple pathways related to microbial infection, such as *Pertussis*, *Yersinia* infection, pathogenic *Escherichia coli* infection, shigellosis, *Salmonella* infection, legionellosis, as well as other signaling pathways, such as ubiquitin mediated proteolysis pathway, NOD-like receptor signaling pathway, cytosolic DNA-sensing pathway, lipid and atherosclerosis signaling pathway, cytosolic DNA-sensing pathway, RIG-I-like receptor signaling pathway, and leishmaniasis pathway (Table 3 and Figure 3D).

### 3.3. Significant and Highly Valued DEGs

**Figure 4:**
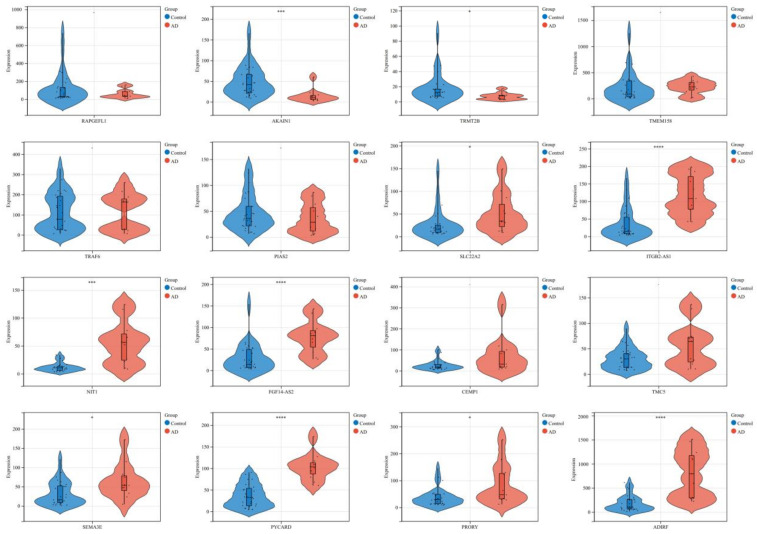
Verification of the DEGs through violin plot. *: *p* < 0.05 (*TRMT2B*, *SLC22A2*, *SEMA3E*, *PRORY*); ***: *p* < 0.001 (*AKAIN1*, *NIT1*); ****: *p* < 0.0001 (*ITGB2-AS1*, *FGF14-AS2*, *PYCARD*, *ADIRF*); -: not significant (*RAPGEFL1*, *TMEM158*, *TRAF6*, *PIAS2*, *CEMP1*, *TMC5*).

The significant DEGs are shown in Figure 4, including ten significant genes (*TRMT2B*, *SLC22A2*, *SEMA3E*, *PRORY*, *AKAIN1*, *NIT1*, *ITGB2*-*AS1*, *FGF14-AS2*, *PYCARD*, *ADIRF*) with *p* value < 0.05, and six non-statistically significant genes (*RAPGEFL1*, *TMEM158*, *TRAF6*, *PIAS2*, *CEMP1*, *TMC5*).

**Figure 5:**
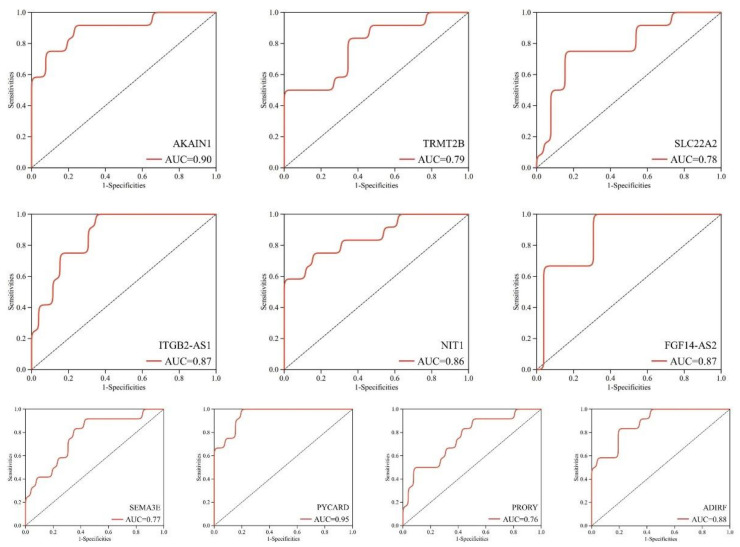
Diagnostic values of DEGs were evaluated by AUC curve in AD. *AKAIN1*: AUC = 0.90; *TRMT2B*: AUC = 0.79; *SLC22A2*: AUC = 0.78; *ITGB2-AS1*: AUC = 0.87; *NIT1*: AUC = 0.86; *FGF14-AS2*: AUC = 0.87; *SEMA3E*: AUC = 0.77; *PYCARD*: AUC = 0.95; *PRORY*: AUC = 0.76; *ADIRF*: AUC = 0.88. The dotted line (diagonal line) indicates an invalid ROC curve, and an AUC value less than 0.5 indicates no diagnostic significance.

In addition, the area under the curve (AUC) analysis under the ROC curve assessed the possible diagnostic utility of these genes (Figure 5). The results showed that the AUC values of these genes were 0.90, 0.79, 0.78, 0.87, 0.86, 0.87, 0.77, 0.95, 0.76 and 0.88, respectively. All of them were greater than 0.75, indicating that they had a potential diagnostic value for AD. Especially for *AKAIN1* and *PYCARD*, AUCs were 0.90 and 0.95, respectively, indicating that they had better therapeutic value.

## 4. Discussion

With the aging global population, the morbidity and mortality rate of AD is increasing, which poses further challenges for human health and social development [4]. The EC is an important region where AD-related neurodegeneration occurs, since it is the first region to be affected before AD spreads to other brain regions. Therefore, early changes in EC can be used as potential early indicators of AD [11,12].

In this study, GSE4757 and GSE21779 gene expression profiles were downloaded from the GEO database to explore the possible candidate genes of AD. Differential gene analysis was performed on AD patients and control subjects. GSE4757 and GSE21779 expression profiles displayed 821 DEGs (572 up-regulated and 249 down-regulated) and 1921 DEGs (958 up-regulated and 963 down-regulated), respectively. In total, 16 DEGs were obtained through the intersection of DEGs from GSE4757 and GSE21779, including 10 up-regulated genes and 6 down-regulated genes. Then, significantly enriched GO terms and KEGG pathways were analyzed to predict the potential biological functions of the DEGs. The KEGG pathway analysis revealed an association with microbial infections, which is in line with the widely discussed microbial hypothesis of the pathological mechanism of AD. Microbes are involved in both maintaining homeostasis of the CNS and can be a potential cause of CNS dysfunction [38,39]. Changes in the composition and function of the microbiota (dysbiosis) may increase the permeability of the intestinal blood barrier and blood brain barrier (BBB) [40,41], which may facilitate the entry of AD pathologically associated gut microbiota products into the CNS, including β-N-methylamino-L-alanine (BMAA), lipopolysaccharides (LPS), and microbial amyloid proteins. This increased BBB permeability can promote neurodegeneration, cognitive impairment, astrogliosis, NFTs accumulation, and brain amyloidosis via the promotion of gut microbiome derived molecules (LPS) and metabolites (short chain fatty acids, SCFA), producing a proinflammatory state, thus, laying the foundation for the pathogenesis of neurodegenerative diseases such as AD [41].

Subsequently, to verify the significant expression verification of the 16 identified DEGs, a ROC curve was used to detect their diagnostic values. The results showed that 10 genes were significant. The AUC values of 10 genes were 0.90, 0.79, 0.78, 0.87, 0.86, 0.87, 0.77, 0.95, 0.76 and 0.88, respectively, indicating that they had a potential diagnostic value for AD. Especially for *AKAIN1* and *PYCARD*, AUCs were 0.90 and 0.95, respectively, indicating that they had better diagnostic value. The identified DEG *PYCARD* is an apoptosis-associated spot-like protein (ASC), which contains the domain (PYD) and a caspase recruitment domain (CARD). The N-terminal PYD of ASC is linked to NOD-like receptors through the interaction of homotypic PYD. The C-terminal is connected to caspase-1 via the interaction of CARD. Caspase-1 also activates the pore-forming gasdermin D, and induces cell death through pyroptosis [42]. PYD and CARD can modulate signaling complexes in apoptotic and inflammatory signaling pathways. *PYCARD* speck formation can activate the inflammasome signaling initiates, so *PYCARD* can also act as the inflammasome complex adaptor protein. Inflammasome activation plays a central role in neurodegenerative diseases such as AD [43].

The disease process of AD, like many diseases, involves complex inflammatory signaling pathways, which cause activation of the innate immune system [44,45]. Neuroinflammation involves a complex set of reactions, including changes in the levels of cellular, molecular, and neuroprotective proteins, the increase in phagocytosis, the aggregation of peripheral immune cells, induction of intracellular signaling pathways, and the release of inflammatory mediators in the brain [46], and the inflammasome-dependent formation of ASC specks in the microglia. These ASC specks are inflammation-driven and bind rapidly to amyloid beta, contributing to increased formation of amyloid-beta oligomers and aggregates that are responsible for the deposition of the pathological amyloid-beta plaques [47,48]. The occurrence of these events may individually, or together, induce the occurrence of neuronal dysfunction and death in AD [49,50,51]. Therefore, the identified *PYCARD* may play an important role in AD. This is consistent with the conclusion of this study, namely that *PYCARD* has a good diagnostic value in AD.

In this study, only the correlation between inflammation related genes in the EC region and AD was explored. Therefore, we have planned a number of follow-up studies. (1) The sample data in other brain regions (such as the hippocampus) will be explored to analyze the potential gene/protein features, and whether they also play an important role in AD neuropathy. (2) In addition, this study will be extended to conduct association analysis between *PYCARD* and neurofibrillary tangles (NFTs) in the EC region to gain a clearer understanding of the mechanism of NFTs formation, which may contribute to the pathogenesis and development of AD. (3) Whether *PYCARD* can be used as a genetic characterization to assess cerebral atrophy of the EC region, and can correlate the degree of brain atrophy observed by magnetic resonance imaging (MRI).

## 5. Conclusions

This work analyzed the DEGs in the EC of AD by computational bioinformatics approaches, and indicated that *AKAIN1* and *TRMT2B* were up-regulated genes, and *SLC22A2*, *ITGB2-AS1*, *NIT1*, *FGF14-AS2*, *SEMA3E*, *PYCARD*, *PRORY*, *ADIRF* were down-regulated genes that might play meaningful roles in AD. In particular, *PYCARD* has the highest diagnostic value and can be employed as an early indicator of AD as an inflammation-related gene. Meanwhile, the enriched pathway revealed several pathways including microorganisms, which may have implications for the microbial hypothesis associated with AD pathology, and may shed new light on the pathogenesis of AD.

## Figures and Tables

**Table 1:** 16 DEGs associated with AD.

DEGs	Count	ID	*p* Value	logFC	Gene	Description
Up_regulated	10	207429_at	0.042	1.884	*SLC22A2*	solute carrier family 22 member 2
		229041_s_at	0.013	2.136	*ITGB2-AS1*	ITGB2 antisense RNA 1
		241395_at	0.022	1.739	*NIT1*	nitrilase 1
		243225_at	0.047	2.123	*FGF14-AS2*	FGF14 antisense RNA 2
		222009_at	0.039	1.559	*CEMP1*	cementum protein 1
		219580_s_at	0.027	1.648	*TMC5*	transmembrane channel like 5
		206941_x_at	0.028	3.083	*SEMA3E*	semaphorin 3E
		221666_s_at	0.048	1.968	*PYCARD*	PYD and CARD domain containing
		221179_at	0.026	1.979	*PRORY*	proline rich, Y-linked
		203571_s_at	0.020	1.741	*ADIRF*	adipogenesis regulatory factor
Down_regulated	6	218657_at	0.020	−1.624	*RAPGEFL1*	Rap guanine nucleotide exchange factor like 1
		243506_at	0.001	−2.416	*AKAIN1*	A kinase (PRKA) anchor inhibitor 1
		1554279_a_at	0.048	−1.434	*TRMT2B*	tRNA methyltransferase 2 homolog B
		213338_at	0.048	−1.780	*TMEM158*	transmembrane protein 158 (gene/pseudogene)
		227264_at	0.031	−2.005	*TRAF6*	TNF receptor associated factor 6
		243624_at	0.040	−1.769	*PIAS2*	protein inhibitor of activated STAT 2

**Table 2:** GO enrichment analysis.

GO Analysis	ID	Description	*p* Value	Gene
BP	GO:0001773	myeloid dendritic cell activation	0.0001	*TRAF6*, *PYCARD*
	GO:0006919	activation of cysteine-type endopeptidase activity involved in apoptotic process	0.001	*SLC22A2*, *PYCARD*
	GO:0042102	positive regulation of T cell proliferation	0.002	*TRAF6*, *PYCARD*
	GO:0002495	antigen processing and presentation of peptide antigen via MHC class II	0.002	*TRAF6*, *PYCARD*
	GO:0002504	antigen processing and presentation of peptide or polysaccharide antigen via MHC class II	0.002	*TRAF6*, *PYCARD*
	GO:0002821	positive regulation of adaptive immune response	0.002	*TRAF6*, *PYCARD*
	GO:0042476	odontogenesis	0.003	*TRAF6*, *CEMP1*
	GO:0043280	positive regulation of cysteine-type endopeptidase activity involved in apoptotic process	0.003	*SLC22A2*, *PYCARD*
	GO:0050671	positive regulation of lymphocyte proliferation	0.003	*TRAF6*, *PYCARD*
	GO:0032946	positive regulation of mononuclear cell proliferation	0.003	*TRAF6*, *PYCARD*
CC	GO:0035631	CD40 receptor complex	0.006	*TRAF6*
	GO:0061702	inflammasome complex	0.010	*PYCARD*
	GO:1902554	serine/threonine protein kinase complex	0.049	*PYCARD*
MF	GO:0031996	thioesterase binding	0.008	*TRAF6*
	GO:0043422	protein kinase B binding	0.008	*TRAF6*
	GO:0070700	BMP receptor binding	0.009	*PYCARD*
	GO:0015562	efflux transmembrane transporter activity	0.010	*SLC22A2*
	GO:0097153	cysteine-type endopeptidase activity involved in apoptotic process	0.011	*PYCARD*
	GO:0005523	tropomyosin binding	0.011	*PYCARD*
	GO:0008381	mechanosensitive ion channel activity	0.012	*TMC5*
	GO:0008656	cysteine-type endopeptidase activator activity involved in apoptotic process	0.012	*PYCARD*
	GO:0015174	basic amino acid transmembrane transporter activity	0.012	*SLC22A2*
	GO:0031435	mitogen-activated protein kinase binding	0.013	*TRAF6*

**Table 3:** KEGG enrichment analysis.

ID	Description	*p* Value	Gene
hsa05133	*Pertussis*	0.001	*TRAF6*, *PYCARD*
hsa05135	*Yersinia* infection	0.003	*TRAF6*, *PYCARD*
hsa04120	Ubiquitin mediated proteolysis pathway	0.003	*TRAF6*, *PIAS2*
hsa04621	NOD-like receptor signaling pathway	0.005	*TRAF6*, *PYCARD*
hsa05130	Pathogenic *Escherichia coli* infection	0.006	*TRAF6*, *PYCARD*
hsa05417	Lipid and atherosclerosis signaling pathways	0.007	*TRAF6*, *PYCARD*
hsa05131	Shigellosis	0.009	*TRAF6*, *PYCARD*
hsa05132	*Salmonella* infection	0.009	*TRAF6*, *PYCARD*
hsa05134	Legionellosis	0.035	*PYCARD*
hsa04623	Cytosolic DNA-sensing pathway	0.038	*PYCARD*
hsa04622	RIG-I-like receptor signaling pathway	0.042	*TRAF6*
hsa05140	Leishmaniasis pathway	0.047	*TRAF6*

## Data Availability

The GSE4757 and GSE21779 gene expression profiles (GPL570: Affymetrix human genome chips U133 Plus 2.0 Array) were downloaded from the Gene Expression Omnibus (GEO) database (http://www.ncbi.nlm.nih.gov/geo/ (accessed on 5 January 2023)).
